# Structure, Morphology and Optical Properties of Chiral *N*-(4-X-phenyl)-*N*-[1(*S*)-1-phenylethyl]thiourea, X= Cl, Br, and NO_2_

**DOI:** 10.3390/molecules15010554

**Published:** 2010-01-26

**Authors:** Werner Kaminsky, Donald Responte, Dan Daranciang, Jose B. Gallegos, Bao-Chau Ngoc Tran, Tram-Anh Pham

**Affiliations:** Department of Chemistry, University of Washington, Seattle, Washington 98195, USA

**Keywords:** aryl ureas, enantiopure crystals, X-ray diffraction, thioamide dimer, non-linear optical properties, Hirshfeld surfaces, OPTACT, dipole-dipole interaction, Bravais-Friedel, Donnay-Harker

## Abstract

Three new enantiopure aryl-thioureas have been synthesized, *N*-(4-X-phenyl)-*N*-[1(S)-1-phenylethyl]thiourea, X= Cl, Br, and NO_2_ (compounds **1**-**3**, respectively). Large single crystals of up to 0.5 cm^3^ were grown from methanol/ethanol solutions. Molecular structures were derived from X-ray diffraction studies and the crystal morphology was compared to calculations employing the Bravais-Friedel, Donnay-Harker model. Molecular packing was further studied with Hirshfeld surface calculations. Semi-empirical classical model calculations of refractive indices, optical rotation and the electro-optic effect were performed with OPTACT on the basis of experimentally determined refractive indices. Compound **3** (space group P 1 (No. 1)) was estimated to possess a large electro-optic coefficient r_333_ of approximately 30 pm/V, whereas **1** and **2** (space Group P 2_1_ (No. 4) exhibit much smaller effects.

## 1. Introduction

Physical features of crystals are governed by their composition and symmetries (Neumann principle: a physical feature possesses at least the symmetry of the material of consideration). In the absence of inversion symmetry, several non-linear optical properties can exist whereas the presence of inversion symmetry prohibits optical rotation or the linear electro-optic effect for example [1]. Within the context of our goal to predict non-linear optical properties from the data provided in molecular crystal structures obtained via X-ray diffraction we were studying closely related chiral compounds for the impact of small differences in chemical composition on the structure-feature relationship.

Crystals will grow in a way that minimizes as much as possible the sum of dipoles of all molecules inside a single unit cell. The same molecule may arrange in different ways to cancel out a net-dipole. Examples of polymorphs of the same chemical compound usually show a minimized dipole charge of the crystal [2]. Geometrical strategies to minimize a total dipole include dimers of a right handed and a left handed molecule of the same chemical composition, related to each other through inversion symmetry. But inversion symmetry inside the unit cell is not possible in case of enantiopure molecules which causes difficulties for some molecules to grow into larger crystals. A residual dipole charge of the crystal, in addition, may cause individuals to grow in form of twins where the dipoles of the twin components cancel out. 

Here we study enantiopure compounds synthesized from the reaction of aryl isothiocyanates with enantiopure α-*S*-methylbenzylamine (aMBA), The products of aMBA when linked to isothiocynates will also be enantiopure. With some odds against our project to obtain useful crystals for the aforementioned reasons, thioureas are also known for the –H-N-C=S functional group to dimerize via the R_2_^2^(8) ring formation, {…H-N-C=S}_2_ [3], structuring the packing of the molecule, minimizing the dipoles, and thereby enhancing crystal growth.

While aryl isothiocyanates have been studied in biological [4] and medicinal applications [5,6], structural or optical properties, and potential optical applications of chiral thioureas remain mostly unexplored. The point groups of compounds presented here are compatible with the aforementioned features that require the absence of inversion symmetry. 

**Scheme 1:**
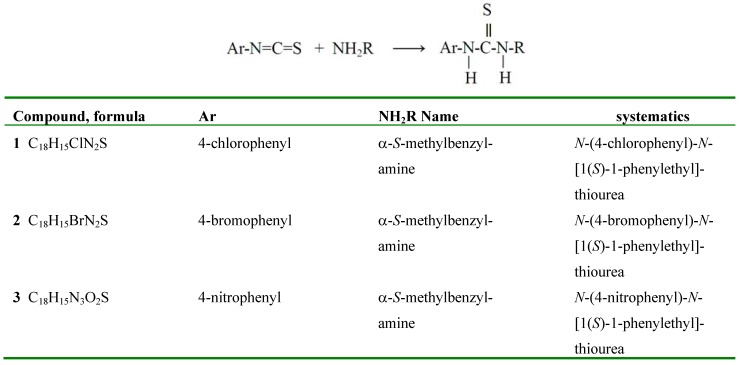
The compounds of this report.

Some similar products from reacting isothiocyanates with alcohols are known, where Ar=H- or NO_2_-phenyl and R=Me, Et, *i*-propyl, *i*-butyl [7,8,9,10], Ar= H-, NO_2_-, C(+O)Me-, Cl-phenyl and R=CH_3 _[11] as well as R = *i*-propyl [12], mostly exhibiting the R_2_^2^(8) dimer, also observed in another series of ureas by us [13,14,15,16,17,18,19,20,21,22,23]. However, this type of dimerization may not apply to all ureas [24]. Summarized in Scheme 1 are three enantiopure ureas **1**-**3**, to the best of our knowledge not previously described in literature, which we will discuss with respect to synthesis, molecular structure, morphology, and non-linear optical properties.

## 2. Results

### 2.1. Synthesis and basic properties

Chemical reactions were based on the general procedure described elsewhere [25]. A stochiometric mixture of isothiocyanate and α-(*S*)-methylbenzylamine was gently stirred in excess of ethanol at 0 °C for 2 hours. The resulting solid product was filtered out and dissolved in 80% ethanol 20% methanol and left for slow evaporation to crystallize. Crystals of compound **3** are very soft and bend easily. Attempts to cut the crystals reveal cleavage on (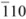). Crystals of **1 **cleave on (010).

### 2.2. Crystal morphology and twinning

Idiomorphically grown crystals were indexed via X-ray diffraction and by comparison with morphologies created with WinXmorph [26,27]. The individual indexing results are complemented with morphologies derived from the Bravais-Friedel, Donnay-Harker model (BFDH) [28,29,30]. 

Morphology predictions with BFDH are closer to the experimental observations in crystals with fewer directional bonds. BFDH predictions for compounds **1** and **2** deviate from the indexed shape significantly which indicates strong directional bonds, whereas compound **3** adds H...O_2_-N hydrogen bonds which dilute the directional character of the N..H-S formation and we observe a far better fit of the predicted morphology (cf. Figure 2). All faces of the indexed twin crystal are observed in the BFDH model, predicting (001) and (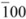) faces to be dominant in **3** and (010) as well as (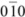) largest in **1** and **2**. If strong polarity is added to the calculation for **3** by which the central distances of (h k l) are extended and those of (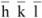 ) are shortened as a simple approach to simulate the symmetry independence of Friedel pairs, the model can be made to exhibit exclusively the observed faces. 

**Figure 1:**
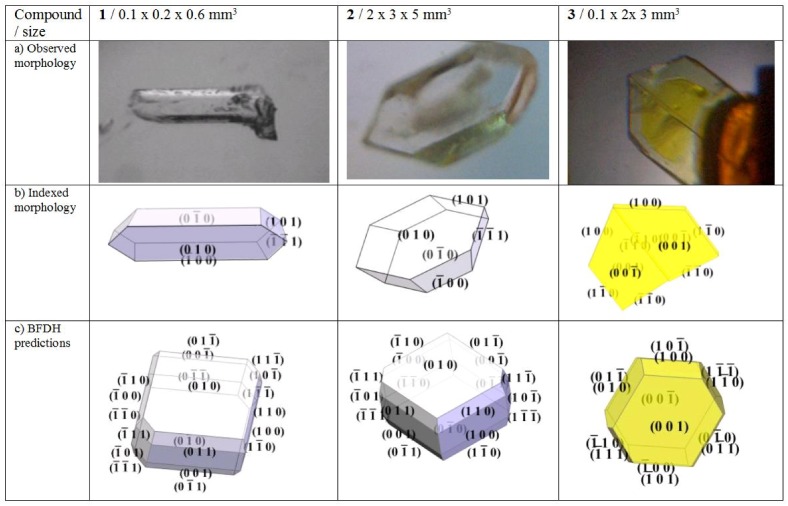
a) Photographs of idiomorphically grown specimen of **1**: *N*-(4-chlorophenyl)-*N*-[1(*S*)-1-phenylethyl]thiourea, **2**: *N*-(4-bromophenyl)-*N*-[1(*S*)-1-phenylethyl]thiourea, **3**: *N*-(4-nitrophenyl)-*N*-[1(*S*)-1-phenylethyl]thiourea. b) indexed morphologies. c) estimated morphologies with the Bravais-Friedel, Donnay-Harker models.

Several individuals of compound **3** were twinned after a twofold twin axis along [110] which eliminates any residual dipole perpendicular to that direction (Figure 1). Compound **1 **grew frequently in the shape of conglomerates. **2 **developed into non-twinned specimen.

### 2.3. X-ray diffraction studies

Crystals measuring 0.2 to 0.5 mm^3^ were mounted on glass capillaries with paratone oil. Diffraction patterns were collected at 130 K on a Nonius Kappa CCD FR590 single crystal X-ray diffractometer, Mo-radiation. Crystal-to-detector distances were 30 mm and exposure times ranged from 20 to 100 seconds per degree for all sets. The scan width was 1–2^o^. The data was integrated and scaled using hkl-SCALEPACK [31]. This program applies a multiplicative correction factor (S) to the observed intensities (I) and has the following form:

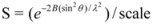
(1)

**Figure 2:**
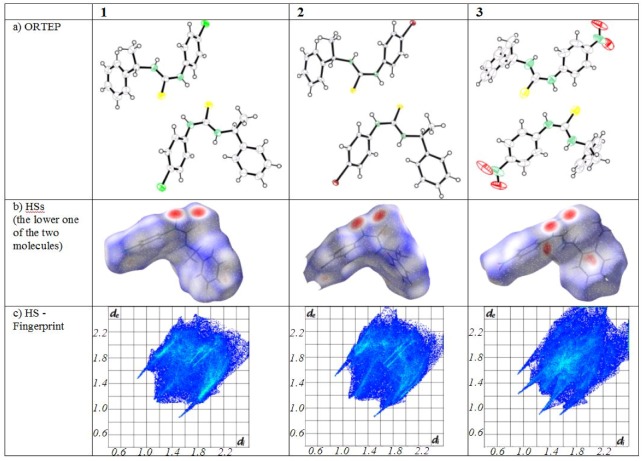
a) ORTEP representations of **1 **- **3** with thermal ellipsoids at the 50% level. b) Hirshfeld surfaces. c) Hirshfeld surface fingerprints of atomic distances.

S is calculated from the scale and the B factor determined for each frame and is then applied to I to give the corrected intensity (I_corr_). Solution by direct methods (SIR97 [32,33] produced nearly complete heavy atom phasing models consistent with the proposed structures. The structures were completed by difference Fourier synthesis with SHELXL97 [34,35].^.^ Scattering factors are from Waasmair and Kirfel [36]. All hydrogen atoms were located using a riding model. All non-hydrogen atoms were refined anisotropically by full-matrix least-squares. Table 1 summarizes the data collection details. ORTEP drawings of the structures for compounds **1**-**3** were rendered with ORTEP3 for Windows (see below, Figure 2) [37]. 

**Table 1:** Crystallographic data and refinement details for crystals **1**–**3**. (*) Compound **3** shows a doubled cell at 130 K with 4 independent molecules, where the cell parameters, **a**, **b**, **c** at room-temperature, presented here, are related to those at 130 K, **A**, **B**, **C**, via the transformation **a** = ½(**A** + **B**), **b** = ½(**B**−**A**), **c** = **C**.

Compound	1	2	3
Formula	C_15_H_15_N_2_ClS	C_15_H_15_N_2_BrS	C_15_H_15_N_3_O_2_S
Formula weight	290.80	335.26	301.36
Crystal system	monoclinic	monoclinic	triclinic
Space group	P2_1_	P2_1_	P1
T/K	130	130	293*
Color / description	colorless needle	colorless plate	yellow plate
Size (mmm^3^)	0.59x0.22x0.12	0.50x0.20x0.14	0.50x0.48x0.05
a/Å	7.4610(3)	7.6500(3)	7.737(3)
b/ Å	24.627(1)	24.645(1)	8.267(3)
c/ Å	7.8620(3)	7.7780(3)	13.616(7)
α/º	90	90	91.43(2)
β/º	100.478(2)	101.113(2)	102.06(2)
γ/º	90	90	112.99(2)
V/Å^3^	1420.5(1)	1438.9(1)	778.5(3)
Z	4	4	2
D_c_/gcm_3	1.360	1.548	1.286
µ(Mo Kα)/mm^-1^	0.403	2.989	0.215
Measured / unique data	4842 / 4842	5864 / 5864	4641 / 4641
Flack enantiopole	-0.2(1)	-0.02(2)	-0.1(2)
R_lin_, No. refined parameters	0.061, 345	0.096, 345	0.088, 381
Observed data, I>2σ(I)	3835	4686	2984
R, obs.; Rw all data	0.0775; 0.2210	0.0672; 0.1730	0.0764; 0.2174
GOOF; compl. (ϑ=25º)	1.111; 99.6	1.065; 99.4	1.026; 96.2

### 2.4. Refractive indices

As light passes from one transparent medium to another, it changes speed and the direction bends as a result of refraction. The degree to which this happens depends on the refractive index of the media along the light polarization, and the angle of incidence. Using a micron scale microscope and the “three height method” [38] which is derived from Snell’s law of refraction, we were able to determine some of the refractive indices in compound **3 **directly in the visible spectral range. The refractive indices are computed approximately from the ratio of distances measured from top to bottom of a crystal plate and from the top to the bottom seen through the (001) crystal section.

The values and directions are approximately 1.61(2) along the a-axis and 1.49(2) perpendicular to that along b* in the projection of the crystal face. 

Conoscopic images suggest that these two are to be extended by a third, bigger value. The material was too soft to prepare a prism or a plate thin enough to determine the third value quantitatively. The eigenmode of the largest refractive index is approximately parallel to the direction of the sulfur atom to the nitro group. 

## 3. Discussion

### 3.1. Thioamide R_2_^2^(8) {…H-N-C=S}_2_ dimer

**Scheme 2:**
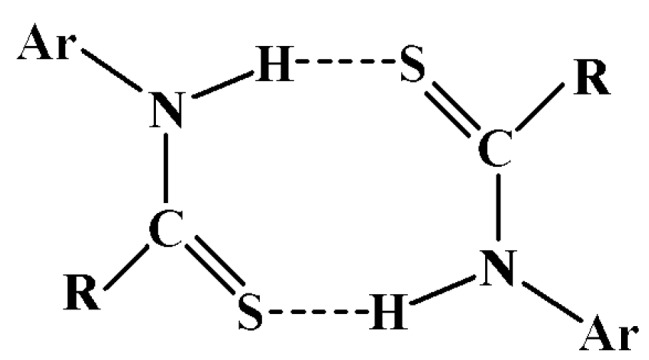
General representation of the thioamide {…H-N-C=S}_2_ dimer formation with R = N-[1(S)-1-phenylethyl] and Ar = 4-Cl-phenyl, 4-Br-phenyl, 4-NO_2_-phenyl.

All structures studied here organize via the thioamide {…H-N-C=S}_2_ dimer formation (Scheme 2). 

Hydrogen bonds are most intuitively studied with Hirshfeld surfaces (HSs, Figure 2, Table 2) which are designed to smoothly enclose almost all available space around a molecule [39,40,41,42] Points ***r*** on a HSs are found where the sum of all spherically approximated electron densities of a molecule at ***r*** is twice the contribution of all atoms in the unit cell at point ***r***. The surface can then be color-coded relative to the distances from points ***r*** to the nearest atoms. 

When choosing a color scheme where red indicates short distances and blue large ones, hydrogen bonds or other close contacts of a molecule to a neighboring one are easily spotted. Figure 2 shows HSs of compounds **1**– **3** in orientation similar to scheme 2. Clearly visible are two ‘red circles’ in close vicinity of sulfur and the corresponding hydrogen bonding nitrogen in each of the shown cases.

HSs can be studied further to establish fingerprint plots where the distance d_i_ to a point ***r*** on the surface to a close atom inside the surface is plotted against the related distance, d_e_ to an outside atom. Figure 2c presents such fingerprint plots for the compounds **1**–**3**. N-H..S hydrogen bonds are contributing to the two outside spikes, H-C bonds occupy the diagonal and two more spikes related to O..H-C hydrogen bonds are found just inside the R_2_^2^(8) formation spikes in **3**.

**Table 2:** Intermolecular hydrogen bonds documenting the thioamide {…H-N-C=S}_2_ dimer formation and NO_2_-H… donor (D) … acceptor (A) interactions in **3**.

	1: H…A, D…A, <(DHA)	2: H…A, D…A, <(DHA)	3: H…A, D…A, <(DHA)
N1-H1N…S2	2.51 Å, 3.345(6) Å, 158.4^o^	2.50 Å, 3.338(9) Å, 159.1^o^	2.59 Å, 3.395(7) Å, 155.2^o^
N3-H3N…S1	2.48 Å, 3.327(6) Å, 162.0^o^	2.50 Å, 3.342(9) Å, 161.6^o^	2.62 Å, 3.400(6) Å, 151.8^o^
N5-H5N…O2	N/A	N/A	2.52 Å, 3.36(1) Å, 166.1^o^

### 3.2. Optical features

#### 3.2.1. Model calculations

**Figure 3:**
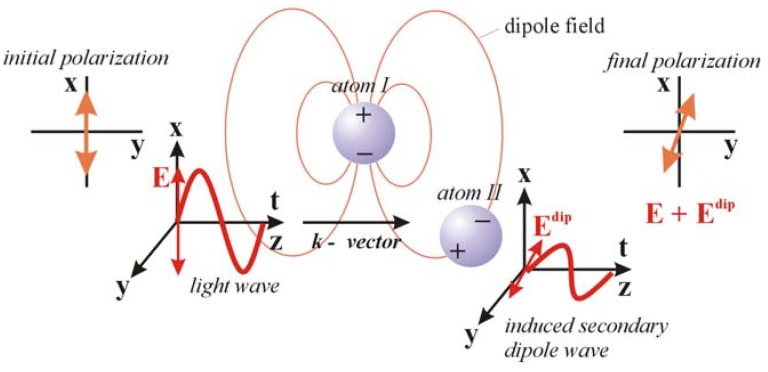
Illustration of the dipole-dipole interaction theory (image from [38], reproduced by permission of The Royal Society of Chemistry). When an initial wave with polarization **E** passes the first atom positioned at (x,0,0) in a Cartesian reference system (**z**//**k**, **k **is the wave vector), a dipole field is created which induces in a second atom at (0,y,z) a secondary dipole field. This field oscillates in a direction different to **E** for y, z ≠ 0. The interference of *all* induced waves with the initial wave (calculated via an Ewald sum) on passing through the crystal leads to a rotation of **E** when the atoms adopt a chiral arrangement.

Properties of purely dispersive chiro-optical properties in crystals may be obtained using the dipole-dipole interaction theory [43,44,45,46,47,48] (Figure 3), which has been applied with success to ionic crystals using OPTACT [49]. Empirical electronic polarizability volumes of the different elements in a structure need to be modelled until the refractive indices calculated with the dipole-dipole model are close to the experimentally derived ones. Starting values for electronic polarizabilities, derived from refractive indices of many different compounds, are tabulated elsewhere [50]. Appendix 1 sketches the theory underlying the OPTACT program used here to calculate refractive indices and optical rotation. 

**Figure 4:**
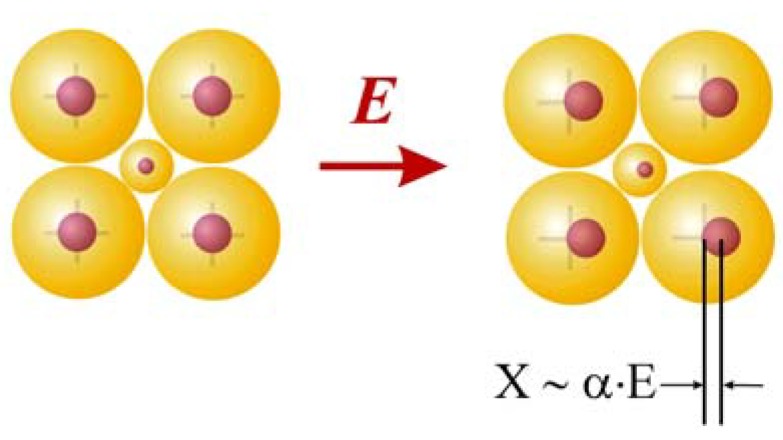
The shift of the nuclei relative to the electron clouds by an external electric field **E** (image from [38], reproduced by permission of The Royal Society of Chemistry). The radius of the clouds indicates the size of the polarizability. The external field induces a small change in the value of the electronic polarizability volumes, but this effect is neglected. Furthermore, in the hard-spheres approximation, the electronic clouds of different atoms are not free to move relative to each other. In a classical picture at optical frequencies, the atom’s nucleus is the center of the light-wave induced vibration of the shell.

While progress has been made in the application of quantum mechanics to the calculation of optical rotation in molecules [51,52], in crystals we chose above more reliable theory that embodies long range interactions and accommodates the periodicity of the crystal lattice. To calculate the higher order optical properties, classical polarizability theory was employed whereby an external electric field displaces the nuclei from the centers of the surrounding electron clouds in proportion to the atomic polarizabilities [53]. Here, the external electric field shifts the atomic nuclei of the k^th^ atom by distance **x** approximated by its electronic polarizability (Figure 4) [54]:

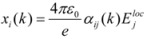
(2)
where *e* is the charge of an electron, *ε*_o_ is the permittivity of free space and *E_j_^loc^* is the local electric field connected with the external field **E***^ext^* via the effective relative dielectric constant *ε*' in the direction of the external field, where a spherical depolarization field (Lorentz-depolarization) is assumed:

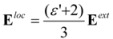
(3)

Atoms with small electronic polarizabilities are less affected by a static electric field than heavy elements [55]. Although it seems to be inappropriate to use electronic instead of ionic polarizabilities, it is observed that if the sum of the molar polarizabilities is increased only by about a factor of three, the dielectric constant tends already to diverge to infinity, as is easily seen from the Clausius-Mossotti relation. Thus we can expect only a relatively small error by this approximation as long as the dielectric constant remains small. 

The aforementioned OPTACT program was used to calculate the optical relative dielectric constant *ε_ij_* with and without the applied field, and with polarization tensor [56] *a_ij_*=*ε_ij_*^-1^, linear electro-optic effects at constant strain r_ijk_ are calculated from:

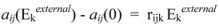
(4)

All tensors were calculated assuming no symmetry at all. Therefore, it was satisfying to discover that the resulting tensors conform to Neumann’s law. 

When choosing electronic polarizabilities to calculate refractive indices and optical rotation we took the published values [50] first, but varying those of O, N, C and H for fitting the experimental refractive indices in **3**. The best set found for elements S, Br, Cl, O, N, C, H was 5.3, 4.4, 3, 2.5, 0.005, 0.09, 0.8 Å^3^, respectively. Table 3 summarizes the extrapolated results [57].

#### 3.2.2. Estimation of optical features

The measured refractive indices and directions in the projection of the (001) crystal face in compound **3** correspond roughly to calculated refractive indices n_β_ and n_α_, see Table 3.

**Table 3:** Calculation of refractive indices, components of corresponding direction cosines (e^o^_x_, e^o^_y_, e^o^_z_), optical rotation and normalized static electro-optic effect at constant strain. Realistic r-coefficients may be obtained from calculated r_ijk_ via r’_ijp_ = ⅓(2+ε_pq_) r_ijq_, ε_pq_ static dielectric constants. Tensor components and directions are related to the physical reference system {**x**, **y**, **z**} via convention **y** // b*, **z** // **c**, **x** = **y** x **z**; **a**, **b**, **c** crystallographic axes.

	1				2				3			
n_α_, **e**^o^	1.513, (0.99,0,0.12)				1.552, (0.98,0,0.21)				1.504, (0.18,-0.88,-0.45)			
n_β_, **e** ^o^	1.606, (0, 1, 0)				1.634, (0, 1, 0)				1.573, (-0.97,-0.07,-0.24)			
nγ, **e** ^o^	1.661, (0.12,0,-0.99)				1.710, (0.21,0,-0.98)				1.767, (0.18,0.45,-0.86)			
Opt. rotation	2.9 0 -5.8				-4.3 0 -9.7				13.7 -10.7 4.8			
	0 -8.4 0				0 -8.0 0				-10.7 -20.0 -21.3			
ρ_(ij)_ (^o^/mm)	-5.8 0 9.1				-9.7 0 -0.1				4.8 -21.3 32.4			
normalized	r_112_	-0.01	r_231_	-0.38	r_112_	-0.12	r_231_	-0.45	r_111_	-0.43	r_221_	2.40
	r_121_	0.07	r_233_	-0.48	r_121_	-0.18	r_233_	-0.35	r_112_	-0.15	r_222_	-1.82
El. Opt. effect	r_123_	-0.44	r_312_	-0.44	r_123_	-0.54	r_312_	-0.44	r_113_	0.11	r_223_	1.82
r’_(ij)k_/ε (pm/V)	r_222_	0.19	r_332_	-0.19	r_222_	0.16	r_332_	-0.16	r_121_	-0.04	r_231_	-1.00
									r_122_	0.22	r_232_	2.08
									r_123_	0.10	r_233_	0.94
									r_131_	-0.24	r_331_	1.27
									r_132_	-0.08	r_332_	0.03
									r_133_	0.04	r_333_	-3.07

In compound **2**, the extinction angle of the largest refractive index in the (010) plane was determined to 11 degrees counter clockwise towards [001] when looking towards the b-axis. The calculated angle was 12.18^o^, in excellent agreement with the observation.

**Figure 5:**
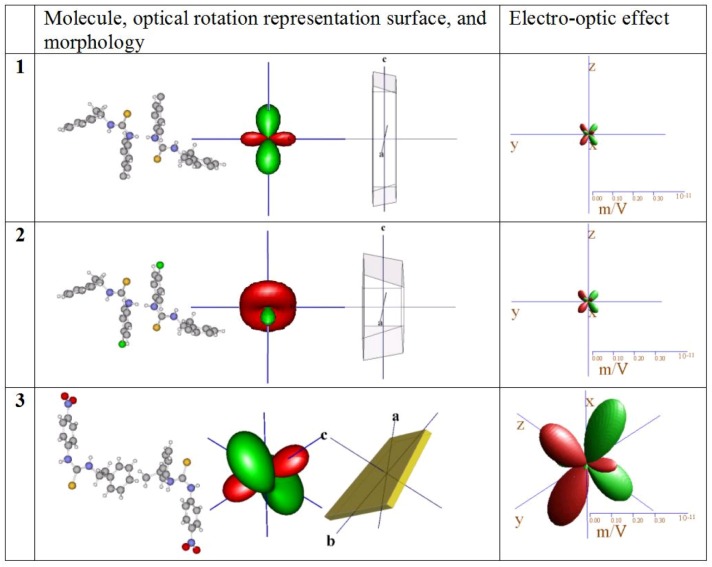
Molecules in similar orientation, representation surface for optical rotation, morphology of typical specimen, and the electro-optic effect, shown to scale to enhance comparison between compounds.

Only weak similarities are observed between the calculated optical rotation of the three substances (Table 3, Figure 5). The coefficient ρ_22_ of optical rotation along the y-axis of the physical reference system is similar of size and negative in **1**, **2** and the equivalent direction in **3** mainly normal to the aryl ring planes. Because there is no symmetry restriction in **3**, we should expect the OR tensor to be different to that of **1** and **2**. Evident is also the effect of the polarizability of the halides in the large differences between **1 **and **2 **even so their atomic structures are almost identical.

Compound **3** promises to be a photonic material of interest as it exhibits large calculated non-linear optical coefficients, is stable at ambient conditions and crystallizes well. It is evident that the orientation of the molecules and the anisotropy of the optical rotation and the electro-optic effect are coupled. For instance, when the molecules of **1 **- **3** are aligned in a similar fashion as in Figure 5, the representation surfaces of the electro-optic effect are also similar in shape and orientation. Assuming a moderate relative dielectric coefficient of about 10, compound **3** could reach a r’_333_ coefficient of 30 pm/V which compares to other non-linear compounds of interest like LiNBO_3_ (r’_333_ = -30.9 pm/V) or KH_2_PO_4_ (r’_123_ =10.3 pm/V) [58].

## Supplementary Data

CCDC 758977-758979 contain the supplementary crystallographic data for this paper. These data can be obtained free of charge *via* www.ccdc.cam.ac.uk/conts/retrieving.html/ (or from the CCDC, 12 Union Road, Cambridge CB2 1EZ, UK; Fax: +44 1223 336033; E-Mail: deposit@ccdc.cam.ac.uk).

## Appendix 1. Dipole-Dipole interaction model

Optical rotation is described by a totally symmetric tensor. Thus, there always exists a reference system in which optical rotation exhibits 222 point group symmetry. Although the two molecules in **3** are very roughly related by a twofold rotation along [110] there is no further clearly defined twofold rotation perpendicular to that direction. In addition, the two molecules are too close to be treated as individual molecules. An attempt to compare single molecule calculations of optical rotation via quantum mechanical methods with the data presented here would be meaningless.

Let an electrical potential, **V, **the Hertz vector potential, built from terms describing the frequency dependent incident wave, the interaction between all atoms in the unit cell and the interaction between all unit cells in the lattice, describe the cumulative effect of the dipole-dipole interactions between all atoms in the crystal lattice acting on an atom *s* in unit cell *l* at a position **r***_s_^l^*:

(A1)

The term in braces has the periodicity of the lattice. The electric field **E**(**r***_s_^l^*) at atom **r***_s_^l^* originating from the dipole waves emanating from all other atoms (point dipoles) in the structure is described by [59]:

(A2)

where **p***_s_^l^* is the electronic polarization at position **r***_s_^l^* and *α_s_* is the electronic polarizability volume of atom *s*. The imaginary part of *A_ss´_* describes the phase shifts that result in radiative interference and optical rotation. The only conditionally convergent summation is decomposed into a Fourier series that can be separated into two absolutely convergent parts, one in real space and the other in reciprocal space according to the Ewald theorem [60]. A new matrix *C_ss_**_´_* is defined from variables describing the electric field **E**(**r***_s_^l^*):

(A3)

(A4)

where *R* is a parameter selected to ensure convergence. The optical rotation *ρ*(**k**) and optical dielectric constants *ε_ij_* are derived as follows:

(A5)

(A6)

where (*e_rij_* = Levi-Civita symbol, **k **= wave vector, *n* = average refractive indices, *v* = unit volume and *δ_ij_* = Kronecker delta). The Levi-Civita operation *e_rij_* accomplishes the cross product between the spatial coordinates of *C_ss_**_′_* and the wave vector.

The rotatory power ρ_ij_ is defined as the *clockwise* rotation of linear polarized light passing through a non-birefringent sample of 1 mm thickness as observed when looking towards the light source. This theoretical model reliably calculates optical rotation on the basis of the interacting forces in inorganic structures. It was further applied successfully to molecular crystals where the interacting fields within a molecule are more significant than those between the molecules [61].
